# Immediate Cervical Muscle Response to Optimal Occlusal Positioning: A Crucial Part of Concussion Risk Management

**DOI:** 10.3390/jcm14248813

**Published:** 2025-12-12

**Authors:** Denise Gobert, Gregg Ueckert, Mark Strickland, Leeda Rasoulian

**Affiliations:** 1 Physical Therapy Department, Texas State University, Round Rock, TX 78665, USA; 2 Ueckert Dentistry, 7030 Village Center Dr, Austin, TX 78731, USA; gregg@brainvault.com; 3 School for Health Sciences, University of St. Augustine, 5401 La Crosse Ave, Austin, TX 78739, USA

**Keywords:** orthotic, concussion risk, muscle strength, muscle endurance, occlusal alignment, mouthguard

## Abstract

**Objectives:** Strong cervical musculature is recognized as a protective factor against sports-related concussions. Evidence suggests that jaw clenching may activate cervical muscles, potentially reducing head acceleration during impact. **Methods:** This observational cohort study examined the immediate effects of a customized interocclusal orthotic (CIO) on cervical muscle performance. Forty-two healthy adults (≥18 years) underwent strength and endurance testing with and without a CIO using a digital pressure gauge and six directional isometric contractions. Descriptive statistics and two-way repeated-measures MANOVA models were applied to evaluate condition effects. **Results:** CIO use produced significant improvements in cervical muscle strength and endurance across all directions compared to non-use. Forward flexion strength increased by 12.96% (*p* < 0.001, ηp^2^ = 0.185), backward extension by 10.34% (*p* = 0.017, ηp^2^ = 0.091), right rotation by 19.03% (*p* < 0.001, ηp^2^ = 0.333) and left rotation by 19.86% (*p* < 0.001, ηp^2^ = 0.353). Endurance gains demonstrated large effect sizes, with flexor endurance improving by 44.57% (*p* < 0.001, ηp^2^ = 0.447). **Conclusions:** Optimized jaw alignment using a customized orthotic can elicit immediate, clinically meaningful enhancements in cervical strength and endurance, suggesting a promising adjunct for concussion risk mitigation in contact sports.

## 1. Introduction

According to the Centers for Disease Control and Prevention (CDC), sports-related concussions (SRCs) affect an estimated 1.6–3.8 million individuals annually in the United States and account for approximately 5–9% of all sports-related injuries [1]. Alarmingly, nearly 70% of emergency department visits for sports and recreation-related traumatic brain injuries (TBIs) involve children under 17 years of age, with boys presenting at twice the rate of girls [1,2]. These injuries occur in both helmeted and non-helmeted sports, with higher incidence during competition compared to practice. Among organized sports, tackle football (63%), wrestling takedowns (59%), and collisions in soccer and basketball (51%) represent the leading causes of concussion in the U.S. [1].

Therefore, clinical research continues to explore strategies to mitigate concussion risk through improved head and neck protection.

### 1.1. Background

Preventing concussion remains a significant challenge for rehabilitation specialists, even for injuries considered “mild.” Current evidence indicates that concussions may involve brain contusions resulting from micro- or macro-traumatic events. Microtrauma tears, commonly referred to as “brain shear,” represent repetitive stretch injuries that can lead to corpus callosum atrophy [3]. These asymmetrical forces applied to the cranium often produce a shearing effect through the falx cerebri, the membrane connecting the brain’s hemispheres [4]. Consequently, the type of impact—rotational versus linear—has emerged as a critical determinant in concussion biomechanics, underscoring the need to strengthen the neck in specific directions to counteract these shear forces or “brain strain” [3,4].

Recognizing the importance of skull stability during impact, the 2022 Amsterdam International Conference on Concussion in Sport recommended mouthguards as protective equipment [5]. Research priorities included examining mandibular alignment and jaw clenching to optimize natural muscle activation for bracing. While some studies report up to a 28% reduction in SRC incidence with mouthguard use, findings remain mixed [6,7]. Moreover, instrumented mouthguard-helmet systems have yielded disappointing results in attenuating rotational and linear forces during real-time monitoring [8].

### 1.2. Cervical Muscle Strength as Related to Concussion Risk

Neck musculature has emerged as a critical intrinsic factor in head stabilization, influencing both sports performance and injury risk [9,10]. Jaw clenching, which activates bilateral temporalis and masseter muscles, has shown promise in reducing head acceleration during contact [11,12,13]. Evidence suggests that increased cervical strength and endurance—facilitating bracing of the head-neck complex—attenuates kinematic responses following impact, thereby lowering concussion risk [10,14,15].

However, findings remain inconclusive. For example, Farley et al. examined neck strength and concussion incidence among 225 professional male rugby players across a season [13]. Thirty concussions occurred in 29 players, yielding an incidence rate of 13.7 per 1000 h played. Greater neck strength was observed mid- and end-season compared to pre-season, and higher extension strength correlated with lower concussion rates. Similarly, Collins et al. reported that a 10% increase in neck extension strength was associated with a 13% reduction in concussion risk (adjusted IRR 0.87; 95% CI, 0.78–0.98) [10]. Collectively, these studies highlight cervical strength and endurance as modifiable risk factors and essential components of conditioning programs, as emphasized by Perillo and Belhassen [14,16].

Additional research confirms that smaller neck circumference and weaker cervical strength significantly increase concussion risk. Collins further noted that each one-pound increase in neck strength reduced concussion odds by 5% (OR = 0.95; 95% CI, 0.92–0.98) [10].

### 1.3. Optimal Jaw Alignment as Related to Cervical Muscle Strength

**Figure 1:**
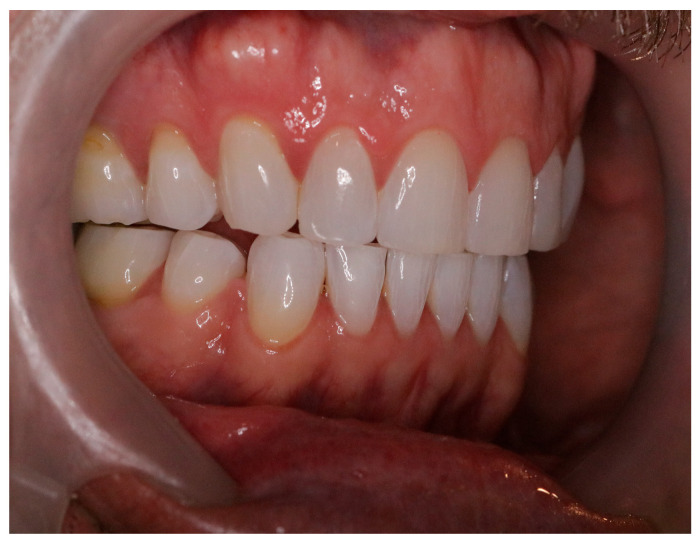
Habitual Jaw Relationship dictated by Maximum Inter-Cusp Position (MIP): the position of the teeth when the posterior teeth are in maximum contact with each other. Jaw muscles need sustained “work” to assume full approximation to accommodate various contact surfaces.

The head, jaw, and neck function as an integrated craniocervical system essential for postural stability and injury prevention. This system includes key muscles of mastication—masseter, temporalis—and cervical stabilizers such as the sternocleidomastoid [17]. In dentistry, the “maximum intercuspal position” (MIP) refers to the habitual position of maximal tooth interdigitation during closure, regardless of condylar position. Essentially, it represents the most stable occlusal contact (see Figure 1).

Research by Fadillioglu and López has demonstrated increased activation of masseter and cervical muscles during jaw clenching, supporting its role in reducing head acceleration during sports contact [18,19,20]. Gage et al. further emphasized that an optimal jaw separation of 3.0–3.5 mm enhances comfort and activation of temporalis, masseter, and sternocleidomastoid muscles during maximal strength efforts in powerlifters [21].

**Figure 2:**
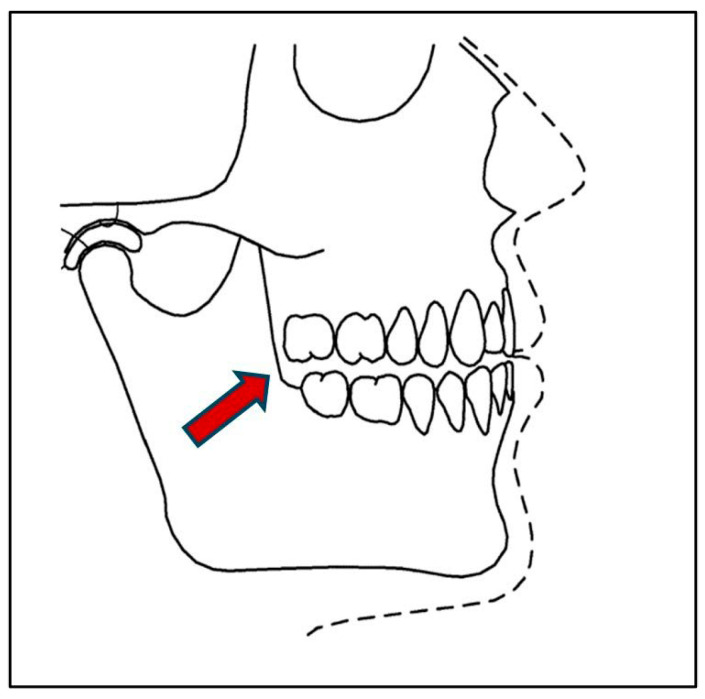
Optimized Jaw Alignment in “Resting” Position: Optimal alignment of the maxilla relative to the mandible after alignment with minimal activation of jaw muscles. The arrow indicates optimal physiological “resting” position space or average interocclusal clearance of approximately 3.0–3.5 mm.

**Figure 3:**
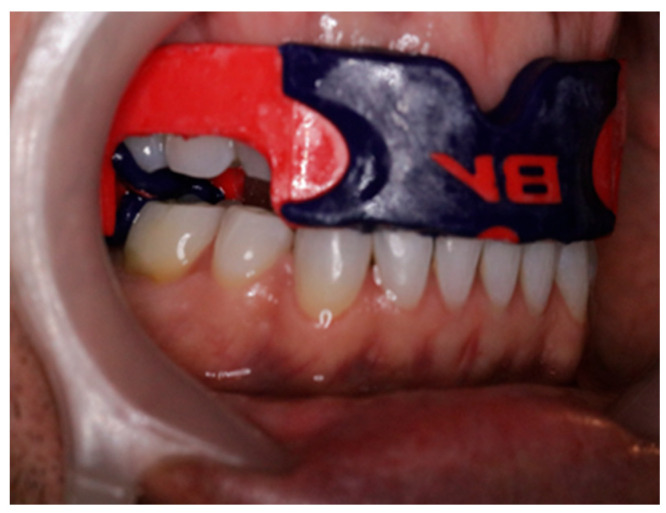
Optimized Jaw Position Supported by Customized Interocclusal Orthotic (CIO): Orthotic creates 6-dimensional stability of the stomatognathic sensory-motor system. An orthotic is in place to allow for optimal approximation of the mandible relative to the maxilla when muscles contract to enhance the position of bracing.

Recent evidence identifies this optimized jaw position—often referred to as the “freeway space”—as critical for promoting efficient static and dynamic motor activity throughout the body [22,23,24,25]. Properly fitted mouth orthotics are therefore advocated to maintain head-neck posture and reduce orofacial injury risk [24,25]. Nonetheless, systematic reviews indicate that further research is needed to clarify the immediate effects of optimal interocclusal alignment on cervical strength and endurance [26] (See Figure 2 and Figure 3).

### 1.4. Purpose

Given this evidence, the present study aimed to examine whether optimal jaw alignment using a customized interocclusal orthotic (CIO) produces immediate changes in cervical muscle force generation.

### 1.5. Research Hypothesis

We hypothesized that cervical musculature would exhibit significantly greater isometric force when participants used a CIO to achieve optimized jaw alignment compared to conditions without the orthotic.

## 2. Materials and Methods

### 2.1. Design

This observational cohort study adhered to the Strengthening the Reporting of Observational Studies in Epidemiology (STROBE) guidelines. Cervical muscle force (kg-force) was measured using a digital pressure gauge (MicroFET^®^ 2, Hoggan Health Industries, Salt Lake City, UT, USA), which demonstrates excellent test–retest reliability (SEM = 0.96–1.71; ICC = 0.94–0.97 at 95% CI). A power analysis (G*Power software 3.1.9.7) indicated that a sample size of 35 would provide 80% power to detect an effect size between 0.4 and 0.8. To ensure adequate power, 40 participants were targeted.

All study procedures, including recruitment and informed consent, were approved by the University’s Institutional Review Board (10323) and conducted in accordance with the Declaration of Helsinki (World Medical Association, www.wma.net, accessed on: 15 September 2025).

Following consent, participants completed a brief medical history to screen for musculoskeletal injuries or recent surgeries that could affect participation. A physical examination assessed vital signs, postural alignment, and active range of motion (AROM) of the cervical spine and shoulders in all planes (Flexion: 45–50°, Extension: 75–80°, Rotation: 65–75°, Lateral Flexion: 35–40°). A Spurling Test was performed to rule out cervical nerve root impingement. Participants then completed two standardized self-report questionnaires to assess neck and shoulder function (see Table 1).

#### 2.1.1. Neck Disability Index (NDI)

The NDI is a validated 10-item questionnaire assessing neck symptoms and functional limitations during daily activities (e.g., work, driving, sleep). Each item uses a 5-point scale, with total scores expressed as a percentage (0–100%), where higher scores indicate greater disability. The NDI demonstrates strong reliability and validity for mechanical neck disorders and requires approximately 3–5 min to complete [27].

#### 2.1.2. Quick Disability of Shoulder & Hand (QuickDASH) Measure

The QuickDASH is an 11-item questionnaire evaluating upper limb function and symptoms during activities such as opening jars or carrying bags. Items use a 5-point scale, with total scores expressed as a percentage (0–100%), where higher scores indicate greater disability. This measure is widely used for musculoskeletal disorders of the upper limbs and demonstrates excellent reliability and validity [28]. Completion time is approximately 3–5 min.

### 2.2. Participants

Forty-two healthy adults (≥18 years) were recruited from the university community via email, flyers, social media, and word-of-mouth.

#### Inclusion and Exclusion Criteria

Inclusion criteria were a normal cervical spine and shoulder, pain-free active range of motion, occlusal bite angle classification types 1–2, no recent head or neck trauma, or oral surgery in the last 6 months. Volunteers were excluded if they had active cervical or shoulder pain, a positive Spurling Test indicating radiculopathy, occlusal bite angle’s classification type 3, limited AROM in the cervical spine or any mandibular (jaw) abnormalities or recent head or neck trauma or oral surgery in the last 6 months, a Neck Disability Index (NDI) score of greater than 20% or a Disability Arm, Shoulder and Hand (QuickDASH) score greater than 40%.

### 2.3. Bite Classification Assessment

A dentist trained in neuromuscular dentistry (per Jankelson principles) [29] assessed occlusal classification using Ackerman and Profitt’s system [30]:

1. Bite Classification Type 1: Upper and lower first molars are aligned with each other so that both upper and lower jaws are the same chin distance. (3 mm overbite with a 3 mm overjet of the upper and lower incisors)
2. Bite Classification Type II: The Lower first molar is aligned more behind the upper first molar on both sides. This is commonly called an “overbite.”
3. Bite Classification Type III: Lower first molar is aligned more anteriorly to the upper first molar on both sides, or the lower teeth and jaw project further forward than the upper teeth and jaw. This is commonly called an “underbite.”

### 2.4. Guided Customized Interocclusal Orthotic (CIO) Proper Insertion

A crockpot containing water was pre-heated to 160 degrees F. When the temperature reached 160 degrees, a new customized interocclusal orthotic mold was placed into the water for 10 s to soften it. Using tongs, the orthotic was then removed from the water and placed on a paper towel to dry before being given to the participant. It was to be self-inserted into the mouth with close instructions as to how to bite down on the orthotic and hold repeatedly 6–8 times over 1 min for proper fitting. The station assessor then guided the participant in the proper fitting of the CIO before allowing the participant to go to the next activity station (Please refer to Figure 3).

### 2.5. Cervical Directional Strength & Endurance Testing

Participants were assessed in a university-based Physical Therapy Outpatient Clinic. Testing stations were used to sequentially test participants through a series of activities. Participants were randomized to start with or without the orthotic in place (please refer to Table 2).

**Table 2:** Physical Performance Testing of Muscle Strength and Endurance. Adapted from Versteegh (2015) [31].

**Figure 4:**
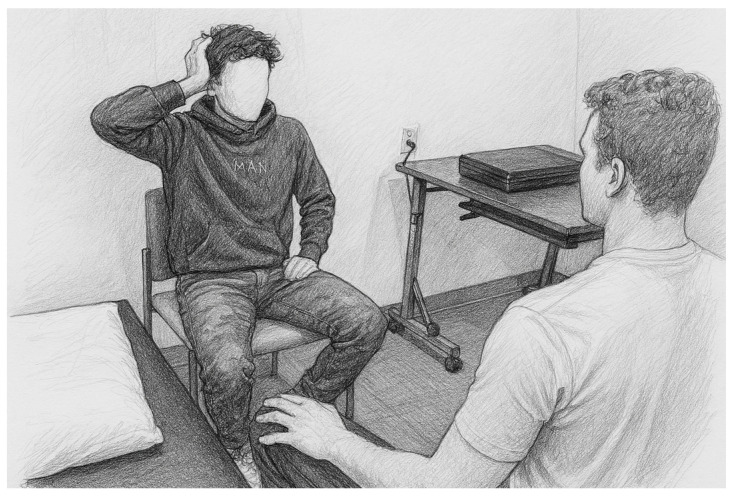
Head-Neck Directional Isometric Muscle Strength Testing (MST): The participant sat in an upright posture in a standard chair with both feet on the floor and was asked to “clench the jaw” while exerting maximum effort in each direction against a hand-held dynamometer microFET^®^ (Hoggan Health Industries, Salt Lake City, UT, USA) keeping the head in a neutral position.

Physical Performance Testing
Hand Grip Strength Test (GST): Participants were asked to perform a grip strength test with each arm using a hand-held dynamometer with the elbow flexed at 90 degrees while sitting in a comfortable, standard chair. Participants were asked to squeeze the handgrip as hard as possible 3 times using the right hand and then repeated the test on the left side. (See Figure 4)
2. Neck Muscle Flexor Endurance Test (MFET): Participants were asked to lie on their back face-up in a comfortable hook-lying position with knees bent on a cushioned plinth. Participants were then asked to lift the head approximately 2.5 cm off the surface in a chin-tucked position as long as possible to fatigue while being timed. This was performed only once. (See Figure 4)
3. Neck Muscle Strength Testing (MST): Finally, each participant was asked to sit in a comfortable chair with both feet on the floor and given a lightweight, handheld dynamometer (MicroFET^®^ 2 force-gauge, Hoggan Health Industries, Salt Lake City, UT, USA) to provide self-generated resistance against 8 head movements for 2–3 s to determine maximum, isometric muscle effort.
Baseline Reference Calibration: Isometric peak force of shoulder horizontal adduction was tested in the participant for baseline calibration. The participant sat comfortably in a standard chair with feet flat on the floor and was instructed to voluntarily and maximally squeeze the MicroFET^®^ (Hoggan Health Industries, Salt Lake City, UT, USA) device between both hands with palms together in front of the body, performing shoulder horizontal adduction. This value was used to determine baseline maximum force to document generated force to be used in opposition to head-neck movements. The participant performed only one peak maximum isometric contraction with and without mouth guard in place.
4. Neck Directional Muscle Testing: After a 3-min rest, isometric neck strength was tested in 6 directions using a neutral head-neck starting position according to the modified protocol described by Versteegh et al., 2015, [31] which demonstrated retest reliability (ICC 0.94–0.97): Head -Neck Forward flexion: resistance applied to the forehead with both hands. Head-Neck Extension: resistance applied to the occiput with both hands. Head-Neck Right & Left Lateral Flexion: resistance applied with the ipsilateral hand just above the ear Head-Neck Right & Left Pure Rotation: resistance applied with the ipsilateral hand along the jaw near the chin with the jaw clenched. Note: Two trials were performed in each direction. (See Figure 4)
5. Standardized Rest Periods: Participants were then allowed to rest for 5–10 min before being asked to insert their customized orthotic in place and repeat all test activities with rest breaks as needed.

### 2.6. Statistical Analysis

Descriptive statistics were used for all variables of interest using IBM^®^ SPSS^®^ version 27 (IBM Inc., Austin, TX, USA). Correlation coefficients were also calculated for all variables using parametric and non-parametric methods according to measurement levels, including demographic data such as age, BMI, and gender. Quality of Life outcomes were addressed using the NDI and DASH scores to describe any possible psychosocial factors or covariates in these analyses. Condition differences were explored using paired t-tests for muscle endurance (in seconds) and strength (kg-force). Two-way repeated measures MANOVA linear models were used to assess the separate and combined influence of main effects of Condition and Position, along with an interaction effect on strength and endurance outcomes as dependent variables. Greenhouse–Giesser corrective estimates were used when violations of sphericity were present. Our alpha level was *p* = 0.5. We also calculated the partial eta squared with ηp^2^ = 0.01 as low, ηp^2^ = 0.06 as medium, and ηp^2^ = 0.14 as large effect sizes.

## 3. Results

Analysis revealed robust and statistically significant differences between the two orthotic conditions across multiple cervical strength and endurance measures. Multivariate testing using Wilks’ Lambda indicated a strong main effect for Condition (F(1,40) = 7.056, *p* < 0.001, ηp^2^ = 0.150) and Gender (F(1,40) = 34.605, *p* < 0.001, ηp^2^ = 0.447), confirming that both factors substantially influenced performance outcomes. Directional isometric strength improved consistently in all six cervical planes—forward flexion, backward extension, bilateral side flexion, and bilateral rotation—with percentage gains ranging from 10.34% to 19.86% when the customized interocclusal orthotic (CIO) was used. These changes were not only statistically significant at *p* < 0.05 but also demonstrated medium to large effect sizes, underscoring the clinical relevance of jaw alignment in enhancing cervical muscle activation. Notably, the most pronounced improvements occurred in rotational strength (up to 19.86%), suggesting a potential mechanism for mitigating rotational head acceleration forces implicated in concussion risk.

In contrast, grip strength remained unchanged (0.19% and 0.95% differences; *p* = 0.480 and *p* = 0.401), highlighting the specificity of the intervention to cervical musculature rather than generalized upper extremity strength. Furthermore, endurance capacity exhibited a dramatic increase, with Neck Flexor Muscle Endurance Test (FMET) times improving by 44.57% under the CIO condition (*p* < 0.001, ηp^2^ = 0.447), representing a large effect size and reinforcing the potential role of optimized occlusal positioning in sustaining cervical stability during prolonged exertion. These findings collectively support the hypothesis that proprioceptive enhancement through customized jaw alignment can produce immediate, meaningful gains in multidirectional cervical strength and endurance, with implications for concussion prevention strategies in contact sports. Please refer to Table 3 for a detailed summary of results.

## 4. Discussion

The present study demonstrated an immediate and statistically significant increase in multidirectional cervical muscle strength and endurance when participants used an optimally positioned customized interocclusal orthotic (CIO) compared to conditions without it. These findings align with prior research by Ceneviz et al., who reported notable changes in EMG activity following mandibular repositioning in healthy subjects, and with Pitteu’s work documenting higher craniocervical muscle activation during soccer heading when a customized mouth orthotic was used (173 N vs. 146 N). Collectively, these results reinforce the concept that jaw alignment can influence neuromuscular performance beyond the masticatory system [17,32].

Our data further supports Tierney’s assertion that multidirectional head stability is essential for mitigating head acceleration events (HAEs), which involve both rotational and linear shear forces commonly implicated in concussion biomechanics [3]. Notably, the most pronounced strength gains in our study occurred in rotational movements (up to 19.86%), suggesting that optimized occlusal positioning may provide a biomechanical advantage in counteracting rotational forces—a mechanism strongly associated with brain strain injury according to Zhan et al. To our knowledge, this is among the first studies to document immediate improvements in cervical rotational strength with a CIO, highlighting a novel avenue for concussion risk reduction strategies [4].

Importantly, these strength gains were specific to cervical musculature, as evidenced by the absence of significant changes in bilateral grip strength. This specificity underscores the targeted effect of jaw alignment on craniocervical stabilization rather than generalized upper extremity performance, adding nuance to prior findings by Zafar et al. [33]. Endurance outcomes were equally compelling, with flexor endurance improving by 44.57% under the CIO condition—a large effect size that mirrors normative trends reported by Domenech et al. and supports Baker et al.’s conclusion that deep neck flexor endurance may predict concussion recovery [8,34].

### Limitations

Several limitations warrant consideration. The sample was restricted to healthy adults aged 18 and older, limiting generalizability to younger athletes or older populations. Additionally, only two conditions—use versus non-use of a CIO—were compared; a non-optimized orthotic condition was not included. Electromyographic data were not collected, precluding analysis of muscle activation patterns, which recent evidence suggests may be critical for concussion risk mitigation. Future research should incorporate EMG measures, explore rate of force development, and compare off-the-shelf mouthguards with customized orthotics to clarify neuromuscular mechanisms. Finally, our sample predominantly represented bite classifications I and II, limiting applicability to individuals with atypical occlusal patterns [29,30].

## 5. Conclusions

This study provides compelling evidence that an optimally positioned customized interocclusal orthotic can produce immediate, clinically meaningful improvements in cervical muscle strength and endurance across multiple planes of motion. These enhancements—particularly in rotational strength and flexor endurance—underscore the potential role of jaw alignment in stabilizing the head-neck complex during high-impact activities. By improving cervical muscle performance, CIOs may offer a practical, non-invasive adjunct to existing concussion prevention strategies in contact sports. Further research is needed to validate these findings across diverse populations, incorporate neuromuscular activation metrics, and examine long-term outcomes. Nevertheless, our results highlight a promising intersection of dental and sports medicine that warrants continued exploration.

## Figures and Tables

**Table 1:** Participant Demographics.

Demographic (Units)	Males (n = 22) Mean (std)	Females (n = 20) Mean (std)	Total (n = 42) Mean (std)
Gender %	52.38	47.62	
Age (years)	30.47 (11.04)	22.76 (2.24)	26.80 (8.94)
BMI (%)	20.20 (11.62)	24.18 (6.60)	22.09 (9.67)
NDI Score (%)	3.41 (4.58)	3.05 (5.90)	3.24 (5.10)
DASH Score (%)	10.55 (6.22)	17.25 (19.66)	13.74 (14.51)
NPRS (units)	0.35 (0.70)	0.45 (0.89)	0.41 (0.80)
Sleep (hours)	7.18 (1.33)	7.48 (0.99)	7.34 (1.16)

NDI = Neck Disability Index; DASH = Disability Assessment of Shoulder & Hand; BMI = Body Mass Index; NPRS = Neurological Pain Rating Scale.

**Table 3:** Results of the Directional Isometric Muscle Strength & Endurance Testing.

Test (Units)	Without CIO	with CIO	Difference %	Effect Size. *p*-Value
1. Forward Flexion (kg-force)	18.58 (8.53)	20.99 (8.78)	12.96 *	η_p_^2^ = 0.185 *p* < 0.001
2. Backward Extension (kg-force)	24.10 (11.68)	26.59 (10.82)	10.34 *	η_p_^2^ = 0.091 *p* = 0.017
3. Right Side-Flexion (kg-force)	20.80 (9.39)	22.96 (8.81)	10.38 *	η_p_^2^ = 0.098 *p* = 0.012
4. Left Side -Flexion (kg-force)	21.13 (9.58)	24.39 (9.76)	15.45 *	η_p_^2^ = 0.310 *p* < 0.001
5. Right Rotation (kg-force)	18.73 (8.95)	22.30 (9.22)	19.03 *	η_p_^2^ = 0.333 *p* < 0.001
6. Left Rotation (kg-force)	18.96 (9.79)	22.73 (10.32)	19.86 *	η_p_^2^ = 0.353 *p* < 0.001
7. Cervical Flexion (in Supine- Seconds)	51.28 (24.34)	74.14 (47.58)	44.57 *	η_p_^2^ = 0.447 *p* < 0.001
8. Right Grip Strength (kg-psi)	109.54 (34.83)	110.58 (34.82)	0.95	η_p_^2^ = 0.005 *p* = 0.401
9. Left Grip Strength (kg-psi)	107.72 (37.31)	107.92 (35.77)	0.19	η_p_^2^ = 0.003 *p* = 0.480

* Alpha level = *p* < 0.05, η_p_^2^: Small = 0.01, Medium = 0.06, Large = 0.14, CIO = Customized Interocclusal Orthotic.

## Data Availability

All data were collected under HIPAA regulations and cannot be publicly reposted, redistributed, or shared.

## Abbreviations

The following abbreviations are used in this manuscript:

BMI	Body Mass Index
CIO	Customized Interocclusal Orthotic
MG	Mouthguard
NDI	Neck Disability Index
NPRS	Neurological Pain Rating Scale
Q-DASH	Quick Disability Assessment of Shoulder & Hand

## References

[1] Quick B.L., Glowacki E.M., Kriss L.A., Hartman D.E. (2021). Raising Concussion Awareness among Amateur Athletes: An Examination of the Centers for Disease Control and Prevention’s (CDC) *Heads Up* Campaign. Health Commun..

[2] Matias-Soto J., Infante-Cano M., García-Muñoz C., Pineda-Escobar S., Martinez-Calderon J. (2024). Concussion Incidence by Type of Sport: Differences by Sex, Age Groups, Type of Session, and Level of Play an Overview of Systematic Reviews with Meta-analysis. J. Orthop. Sports Phys. Ther..

[3] Tierney G. (2021). Concussion biomechanics, head acceleration exposure and brain injury criteria in sport: A review. Sports Biomech..

[4] Zhan X., Li Y., Liu Y., Domel A.G., Alizadeh H.V., Raymond S.J., Ruan J., Barbet S., Tiernan S., Gevaert O. (2021). The relationship between brain injury criteria and brain strain across different types of head impacts can be different. J. R. Soc. Interface.

[5] Daneshvar D.H., Baugh C.M., Nowinski C.J., McKee A.C., Stern R.A., Cantu R.C. (2011). Helmets and Mouth Guards: The Role of Personal Equipment in Preventing Sport-Related Concussions. Clin. Sports Med..

[6] Knapik J.J., Hoedebecke B.L., Rogers G.G., Sharp M.A., Marshall S.W. (2019). Effectiveness of Mouthguards for the Prevention of Orofacial Injuries and Concussions in Sports: Systematic Review and Meta-Analysis. Sports Med..

[7] Quigley K.G., Hopfe D., Fenner M., Pavilionis P., Owusu-Amankonah V., Islas A., Murray N.G. (2024). Preliminary Examination of Guardian Cap Head Impact Kinematics Using Instrumented Mouthguards. J. Athl. Train..

[8] Baker M., Quesnele J., Baldisera T., Kenrick-Rochon S., Laurence M., Grenier S. (2019). Exploring the role of cervical spine endurance as a predictor of concussion risk and recovery following sports related concussion. Musculoskelet. Sci. Pract..

[9] Chavarro-Nieto C., Beaven M., Gill N., Hébert-Losier K. (2021). Neck strength in Rugby Union players: A systematic review of the literature. Physician Sportsmed..

[10] Collins C.L., Fletcher E.N., Fields S.K., Collins C.L., Fletcher E.N., Fields S.K., Kluchurosky L., Rohrkemper M.K., Comstock R.D., Cantu R.C. (2014). Neck Strength: A Protective Factor Reducing Risk for Concussion in High School Sports. J. Prim. Prev..

[11] Garrett J.M., Mastrorocco M., Peek K., van den Hoek D.J., McGuckian T.B. (2023). The Relationship Between Neck Strength and Sports-Related Concussion in Team Sports: A Systematic Review with Meta-analysis. J. Orthop. Sports Phys. Ther..

[12] Raquel G., Namba E.L., Bonotto D., Rosa E.A.R., Trevilatto P.C., Machado M.Â.N., Vianna-Lara M.S., Azevedo-Alanis L.R. (2017). The use of a custom-made mouthguard stabilizes the electromyographic activity of the masticatory muscles among Karate-Dō athletes. J. Bodyw. Mov. Ther..

[13] Farley T., Barry E., Sylvester R., Medici A.D., Wilson M.G. (2022). Poor isometric neck extension strength as a risk factor for concussion in male professional Rugby Union players. Br. J. Sports Med..

[14] Perillo L., Femminella B., Farronato D., Baccetti T., Contardo L., Perinetti G. (2010). Do malocclusion and Helkimo Index ≥5 correlate with body posture?. J. Oral Rehabil..

[15] Rotto T., Kraus E., Fredericson M. (2020). A Neck Strength Training Protocol in High School Football Players for Concussion Risk Reduction. Orthop. J. Sports Med..

[16] Belhassen S., Mat Q., Ferret C., Clavel R., Renaud B., Cabaraux P. (2023). Post-Traumatic Craniocervical Disorders from a Postural Control Perspective: A Narrative Review. Brain Neurorehabilit..

[17] Ceneviz C., Mehta N.R., Forgione A., Sands M.J., Abdallah E.F., Lobo Lobo S., Mavroudi S. (2006). The Immediate Effect of Changing Mandibular Position on the EMG Activity of the Masseter, Temporalis, Sternocleidomastoid, and Trapezius Muscles. CRANIO^®^.

[18] Fadillioglu C., Kanus L., Möhler F., Ringhof S., Hellmann D., Stein T. (2022). Influence of Controlled Stomatognathic Motor Activity on Sway, Control and Stability of the Center of Mass During Dynamic Steady-State Balance—An Uncontrolled Manifold Analysis. Front. Hum. Neurosci..

[19] Fadillioglu C., Kanus L., Möhler F., Ringhof S., Hellmann D., Stein T. (2023). Effects of jaw clenching on dynamic reactive balance task performance after 1-week of jaw clenching training. Front. Neurol..

[20] López Paños R., Ortiz-Gutiérrez R.M., Chana Valero P., Felipe Concepción E. (2019). Valoración del control postural y del equibrio en personas con trastornos temporomandibulares: Revisión sistemática. Rehabilitación.

[21] Gage C.C., Huxel Bliven K.C., Bay R.C., Sturgill J.S., Park J.H. (2015). Effects of mouthguards on vertical dimension, muscle activation, and athlete preference: A prospective cross-sectional study. Gen. Dent..

[22] Różańska-Perlińska D., Jaszczur-Nowicki J., Rydzik Ł., Perliński J., Bukowska J.M. (2023). Changes in Gait Parameters and the Podal System Depending on the Presence of a Specific Malocclusion Type in School-Age Children. J. Clin. Med..

[23] Sliwkanich L., Ouanounou A. (2021). Mouthguards in dentistry: Current recommendations for dentists. Dent. Traumatol..

[24] Sakaguchi K., Mehta N.R., Maruyama T., Correa L.P., Yokoyama A. (2023). Effect of masticatory movements on head and trunk sways, and sitting and foot pressure distributions during sitting position. J. Oral Rehabil..

[25] Ohnmeiß M., Kinzinger G., Wesselbaum J., Korbmacher-Steiner H.M. (2014). Therapeutic effects of functional orthodontic appliances on cervical spine posture: A retrospective cephalometric study. Head Face Med..

[26] Mason R., Franklin H., Grant P., Role E. (2020). The importance of the freeway space in orofacial myofunctional therapy. Int. J. Orofac. Myol. Myofunct. Ther..

[27] Vernon H., Mior S. (1991). The neck disability index: A study of reliability and validity. J. Manip. Physiol. Ther..

[28] Gummesson C., Ward M.M., Atroshi I. (2006). The shortened disabilities of the arm, shoulder and hand questionnaire (Quick DASH): Validity and reliability based on responses within the full-length DASH. BMC Musculoskelet. Disord..

[29] Jankelson B. (1979). Neuromuscular aspects of occlusion. Effects of occlusal position on the physiology and dysfunction of the mandibular musculature. Dent. Clin. N. Am..

[30] Ghodasra R., Brizuela M. (2025). Orthodontics, Malocclusion. StatPearls [Internet].

[31] Versteegh T., Beaudet D., Greenbaum M., Hellyer L., Tritton A., Walton D. (2015). Evaluating the Reliability of a Novel Neck-Strength Assessment Protocol for Healthy Adults Using Self-Generated Resistance with a Hand-Held Dynamometer. Physiother. Can..

[32] Pitteu C., Lepère P., Poisson P., Guillaud E., Doat E., Glize B., Dehail P., Cassoudesalle H. (2025). A custom-made mouthguard reduces head acceleration during soccer heading and prevents acute electrophysiological and cognitive changes in amateur male players. EBioMedicine.

[33] Zafar H., Alghadir A.H., Iqbal Z.A., Iqbal A., Anwer S., Alnahdi A.H. (2019). Influence of different jaw positions on dynamic balance using Y-balance test. Brain Behav..

[34] Domenech M.A., Sizer P.S., Dedrick G.S., McGalliard M.K., Brismee J.M. (2011). The Deep Neck Flexor Endurance Test: Normative Data Scores in Healthy Adults. PM Rehabil..

